# Mating System and Fine-Scale Spatial Genetic Structure of the Tropical Epiphytic Orchid *Rhynchostylis gigantea*

**DOI:** 10.3390/ijms27188176

**Published:** 2026-09-14

**Authors:** Wen-Chang Li, Zhi-Heng Chen, Hao-Tian Zhong, Ming-Xun Ren, Zhe Zhang, Xi-Qiang Song

**Affiliations:** 1 Key Laboratory of Genetics and Germplasm Innovation of Tropical Special Forest Trees and Ornamental Plants (Ministry of Education), School of Tropical Agriculture and Forestry, Hainan University, Danzhou 571700, China; lwc2280637848@gmail.com (W.-C.L.); 18982783311@163.com (Z.-H.C.); stiggg01@gmail.com (H.-T.Z.); 2 Hainan Institute of National Park, Haikou 570203, China; 3 Center for Terrestrial Biodiversity of the South China Sea, Haikou 570228, China; renmx@hainanu.edu.cn; 4 School of Breeding and Multiplication (Sanya Institute of Breeding and Multiplication), Hainan University, Sanya 572024, China

**Keywords:** *Rhynchostylis gigantea*, epiphytic orchid, fine-scale spatial genetic structure, gene flow, parentage analysis, habitat fragmentation

## Abstract

Habitat fragmentation caused by human activities threatens plant genetic diversity, but the mechanisms shaping gene flow and spatial genetic structure in epiphytic orchids remain poorly understood. Here, we investigated the genetic structure and gene flow patterns of the epiphytic orchid *Rhynchostylis gigantea* in a human-modified landscape on Hainan Island, China, using SNP markers. Based on 2005 high-quality SNPs generated via double-digest restriction site-associated DNA sequencing (ddRADseq) from 275 individuals, we assessed genetic diversity, population differentiation, and fine-scale spatial genetic structure (FSGS) and further explored mating patterns through parentage analysis of 100 F1 offspring. The adult population maintained moderate genetic diversity (*H_o_* = 0.243, *F_IS_* = 0.082), whereas offspring cohorts showed stronger heterozygote deficiency (*F_IS_* = 0.198), suggesting that high contemporary geitonogamous selfing may be counterbalanced by post-zygotic selective mortality during early life stages prior to adult recruitment. The two adult subpopulations separated by agricultural fields exhibited significant genetic differentiation (*Φ_PT_* = 0.131, *p* = 0.001), indicating reduced gene exchange connectivity caused by landscape fragmentation. Significant FSGS was detected across the population and subpopulations, with three-dimensional spatial analyses revealing the influence of both spatial distance and host-tree distribution. Parentage analysis revealed a high rate of geitonogamy and a pattern in which single pollen donors fertilized multiple flowers on the same recipient plant. This indicates that pollinator movement is highly restricted, leading to localized mating within immediate flower clusters. Our results demonstrate that the genetic structure of *R. gigantea* is shaped by the combined effects of mixed mating systems, limited seed dispersal, host-tree dependence, and habitat fragmentation. Conservation of epiphytic orchids should therefore integrate the protection of orchid populations, host trees, and landscape connectivity to maintain long-term genetic diversity.

## 1. Introduction

Tropical rainforests represent one of the most biodiversity-rich ecosystems on Earth. However, driven by socioeconomic development, extensive forest clearing and land-use conversion continue to occur worldwide. These processes inevitably result in the reduction and fragmentation of forest habitats, transforming formerly continuous forest landscapes into isolated patches [1]. Such spatial isolation not only alters the distribution patterns of plant communities but also directly affects gene exchange among plant populations and their spatial genetic structure [2,3]. For plant species that rely on pollen and seed dispersal to maintain genetic connectivity, reduced gene flow may increase the risks of inbreeding depression and genetic drift, ultimately compromising population adaptive potential [4,5]. Therefore, under the accelerating pressures of forest loss and habitat fragmentation, understanding the role of gene flow in maintaining plant population dynamics and genetic diversity has become a central issue in conservation biology and evolutionary ecology.

As one of the largest families of angiosperms worldwide, Orchidaceae exhibits unique characteristics of gene flow that differ from those of many other plant groups [6]. Orchid seeds are characterized as “dust-like” seeds, which theoretically enable long-distance dispersal by wind [7]. However, increasing evidence indicates that seed dispersal in orchids is constrained by multiple ecological and biological factors [6,8]. Moreover, orchid seeds lack endosperm, and their germination is entirely dependent on the establishment of symbiotic associations with mycorrhizal fungi, which provide essential nutrients required for germination and early seedling development. Therefore, mycorrhizal fungi play a critical role in orchid mineral nutrition, particularly during the seedling stage, when they are key determinants of orchid establishment and survival [9,10,11]. Because the enrichment and availability of suitable mycorrhizal fungi are often associated with adult orchid individuals, which function as localized fungal reservoirs or “nurse sites”, orchid seed germination frequently occurs in close proximity to maternal plants [6,8]. This spatially restricted recruitment pattern can facilitate the formation of fine-scale spatial genetic structure (FSGS) within orchid populations [12].

Pollen flow represents a critical component of plant gene flow, not only determining the extent of genetic exchange among populations but also profoundly influencing plant genetic structure and adaptive potential [13]. Although some orchid species are capable of reproducing through self-pollination [14], the majority of orchids rely on pollinators for sexual reproduction, and their reproductive success and patterns of gene flow are largely determined by pollinator behavior and ecological interactions [15]. Based on their pollination mechanisms, orchids can be broadly categorized into two major groups: deceptive pollination and reward-based pollination [16]. Deceptive pollination is a prominent characteristic of Orchidaceae, with approximately one-third of orchid species providing no floral rewards such as nectar or pollen. Instead, these species attract pollinators through deceptive strategies, including floral mimicry, sexual deception, and the manipulation of floral traits such as shape and coloration [6,17]. Such strategies often result in shorter residence times of pollinators on individual plants and increase movement distances among plants, thereby facilitating long-distance pollen-mediated gene flow. In contrast, reward-based pollination attracts pollinators by providing resources such as nectar, increasing visitation frequency within local areas. Although this strategy can enhance reproductive success, it may also promote geitonogamous pollination and localized genetic exchange, thereby increasing the potential risk of inbreeding depression [5]. Therefore, understanding the pollination strategy and potential pollinator-mediated pollen transfer is important for interpreting gene flow patterns in orchids.

Nearly 70% of orchid species are epiphytes [18], and this life form is considered one of the most important evolutionary innovations in Orchidaceae, with profound impacts on their survival, speciation, dispersal, and diversification [19]. However, current studies on gene flow in orchids have largely focused on terrestrial orchids, particularly species inhabiting forest floors or herbaceous layers [2,15,20,21]. In contrast, gene flow patterns in epiphytic orchids remain comparatively poorly understood. Because epiphytic orchids inhabit three-dimensional environments, including tree trunks, branches, and canopies, their gene flow processes are jointly influenced by host tree distribution patterns, forest structure, and microenvironmental conditions within the canopy [12,22]. Consequently, epiphytic orchids may exhibit gene flow patterns distinct from those of terrestrial orchids, and their population genetic structures are likely to be more difficult to predict.

First, spatial positioning may generate differences between horizontal and vertical genetic structures [23]. Horizontal structure may arise when individuals growing on the same branch exhibit higher genetic relatedness, whereas vertical structure may occur when individuals occupying different branches but located within the same vertical plane show closer genetic relationships [22,23]. Second, the height at which seeds are released may influence their dispersal distance and colonization range. Finally, founder number and colonization origin may also play important roles in shaping genetic structure. If each host tree is colonized by only one or a few founder individuals, genetic structure may emerge at the tree level (tree-scale SGS), while genetic relatedness among individuals inhabiting different host trees may remain relatively low [22]. In addition, epiphytic orchids often exhibit sequential colonization patterns, whereby offspring gradually establish on neighboring host trees over time [22]. Therefore, within a population, epiphytic orchids occupying adjacent host trees may exhibit closer genetic relationships and are more likely to form spatial genetic structures.

Understanding gene flow in epiphytic orchid populations therefore requires consideration not only of horizontal dispersal but also of potential vertical dispersal processes. Moreover, within epiphytic habitats, mycorrhizal fungi are not only essential for seed germination and seedling establishment but may also connect orchid individuals occupying different spatial locations through shared mycorrhizal networks extending across host tree surfaces [24]. Consequently, even when seeds do not land near maternal plants, they may still have a relatively high probability of successful germination through access to shared fungal networks. This suggests that seeds derived from fruits produced through short-distance pollination may, after dispersal, successfully establish away from maternal plants via mycorrhizal networks, thereby partially weakening the spatial clustering effect caused by restricted pollen flow. However, this mechanism is highly dependent on the availability and continuity of suitable host trees.

For epiphytic orchids, the presence of host trees is fundamental to population establishment and long-term persistence due to the inherent constraints imposed by their growth habit. Epiphytic orchid species associated with different host trees often exhibit significant differences in survival, growth rates, and reproductive performance, which directly contribute to variation in population growth rates [25]. Research has demonstrated that *Bombax ceiba* is a true umbrella tree species, providing structural microhabitats and resources for a diverse range of animals and plants [26]. Owing to its tall trunk, sparse canopy foliage, and other structural characteristics, the deciduous habit and relatively open canopy of *B. ceiba* may create more exposed bark microhabitats, potentially influencing the establishment of epiphytic orchids. For example, in regions such as India and Nepal, orchids represent one of the dominant groups of epiphytic plants inhabiting *B. ceiba* trees [27,28].

*B. ceiba* flowers in early spring, producing abundant flowers before leaf emergence. Its nectar and fleshy petals provide important seasonal food resources for a variety of mammals, birds, and insects. In southern India, *B. ceiba* has been observed to provide food resources or habitat for more than 40 animal species [26]. Such fauna-mediated activity on host trees may deposit organic excreta and bark debris onto tree surfaces, providing supplemental organic nutrients and potentially introducing diverse microbial and fungal propagules. Although arboreal animals are known to transport various fungal spores—such as arbuscular mycorrhizal fungi associated with forest trees—whether fauna-mediated zoochory facilitates the dispersal of specific orchid mycorrhizal fungi (OMF, mainly *Basidiomycota*) across host trees remains an intriguing hypothesis that warrants future investigation [29].

Anthropogenic land-use changes continue to contribute to the continuous reduction and degradation of orchid habitats. However, within the Hainan Tropical Rainforest National Park, several traditional villages inhabited by Li and Miao ethnic communities have preserved unique forest patches due to traditional customs and cultural beliefs, particularly through totem worship. These patches, commonly known as “fengshui forests”, are dominated by tree species such as Moraceae members (e.g., *Antiaris toxicaria*, *Ficus altissima*, and *Ficus virens*), *B. ceiba*, coconut (*Cocos nucifera*), and mango (*Mangifera indica*). They represent a traditional lifestyle characterized by long-term human–nature coexistence and provide a typical example of island tropical indigenous settlements that buffer local ecosystems.

Across western Hainan, indigenous Li and Miao biocultural traditions—such as those famously documented in Changjiang County (widely known as the ‘hometown of kapok’)—have historically safeguarded ancient *Bombax ceiba* trees through cultural veneration and their traditional use in Li brocade weaving [30]. Sharing this identical cultural reverence and biocultural setting, remnant ancient *B. ceiba* stands are also preserved within the agricultural matrix of neighboring Dongfang City in western Hainan. These scattered ancient trees provide vital refuges for epiphytic orchids, including *Rhynchostylis gigantea*, to persist within human-modified landscapes.

*R. gigantea* is a typical drought-tolerant epiphytic orchid that commonly grows on the trunks and lateral branches of large trees, including *B. ceiba*, *M. indica*, and *Bischofia javanica*. Previous field observations in Hainan indicate that *R. gigantea* is a reward-producing orchid that primarily relies on generalist hymenopterans—carpenter bees (*Xylocopa* spp.)—for cross-pollination while maintaining self-compatibility [31].

**Figure 1:**
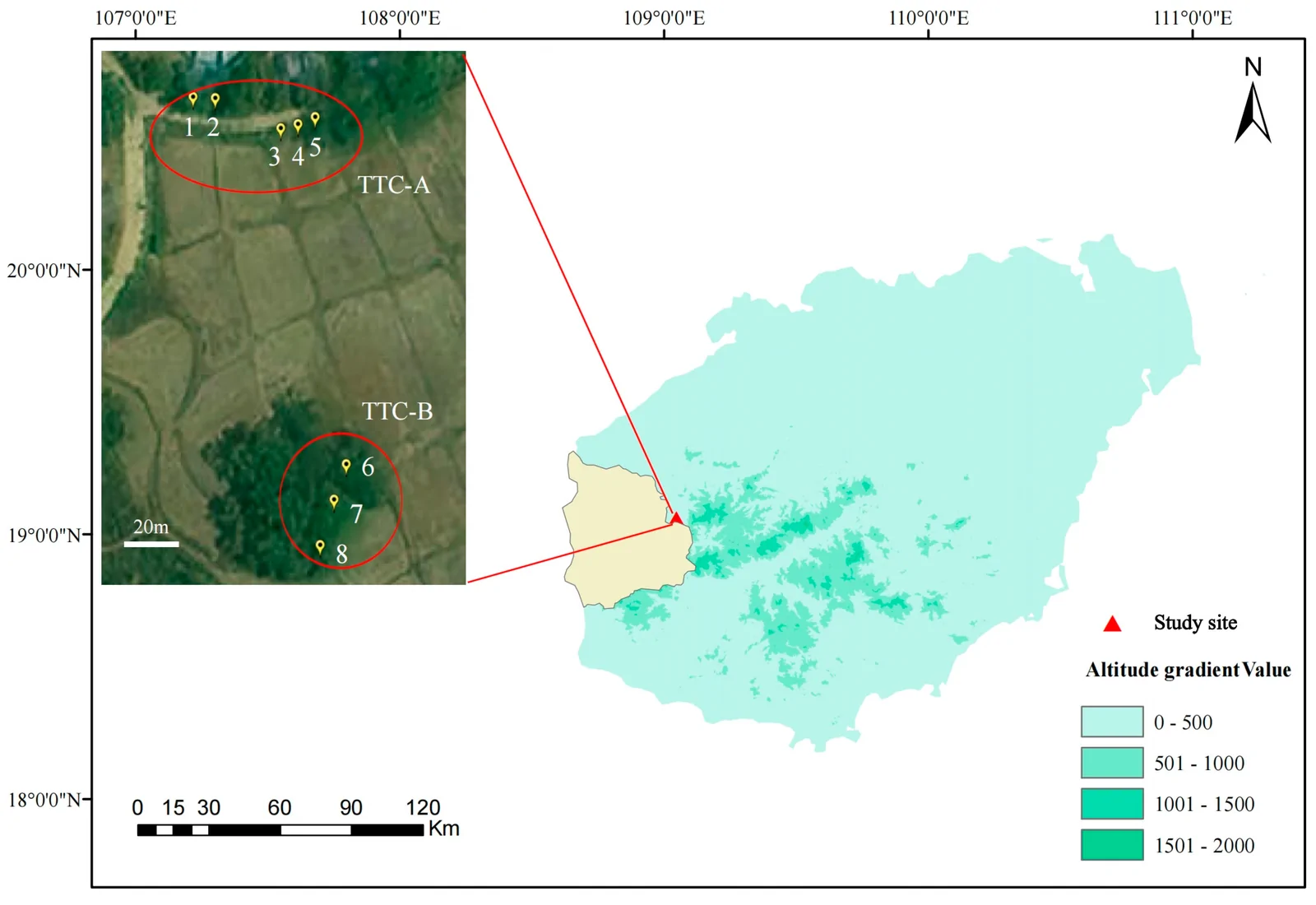
The research area for *R. gigantea* can be divided into two populations, TTC-A and TTC-B. All *R. gigantea* are epiphytic on eight *Bombax ceiba*. The red circles in the figure represent the division of the two subpopulations, and the numbers are the IDs of *Bombax ceiba* trees.

Here, we investigated the gene flow and spatial genetic structure of *R. gigantea* in Jiangbian Township, Dongfang City (108°56.671432’ E, 18°54.061295’ N), where historical *B. ceiba* host trees remain embedded within an agricultural matrix. The two *R. gigantea* subpopulations investigated in this study were separated by an agricultural field measuring approximately 60 × 120 m (Figure 1). Specifically, we hypothesized that: (1) the agricultural matrix restricts gene exchange between subpopulations, contributing to elevated genetic differentiation; (2) the epiphytic habit, spatial distribution of host trees, and limited effective seed dispersal generate tree-level fine-scale spatial genetic structure; and (3) pollinator foraging behavior promotes localized mating and geitonogamy. Given that *R. gigantea* is a nationally protected wild orchid facing population decline due to historical over-harvesting and host-tree loss in fragmented agricultural landscapes, answering these questions provides essential empirical baselines for maintaining host-tree connectivity and guiding targeted germplasm conservation and habitat restoration.

## 2. Results

### 2.1. DNA Extraction and SNP Calling

Genomic DNA was extracted from 275 *R. gigantea* individuals using a modified CTAB method. Agarose gel electrophoresis (1.2%) revealed moderate variation in DNA quality among samples; however, the majority of samples exhibited clear major bands and met the requirements for subsequent library construction. Following sequencing, quality control, and molecular marker development, a total of 2005 high-quality single nucleotide polymorphisms (SNPs) were retained for subsequent analyses.

### 2.2. Genetic Diversity

Genetic diversity indices were assessed in two adult populations (TTC-A and TTC-B) and a separate offspring population of *R. gigantea* (Table 1). Across the total adult sample (N = 175), the average Shannon’s information index (*I*), observed heterozygosity (*H_o_*), and expected heterozygosity (*H_e_*) were 0.407, 0.243, and 0.265, respectively, with an inbreeding coefficient (*F_IS_*) of 0.082, indicating a slight heterozygote deficiency at the population level. Within the TTC-A population (N = 153), genetic diversity was relatively higher (*I* = 0.410), with *H_o_* (0.245) lower than *H_e_* (0.265), resulting in a positive inbreeding coefficient *F_IS_* (0.088), which indicates a slight deficiency of heterozygotes. In contrast, TTC-B (N = 22) exhibited lower diversity (*I* = 0.404, *H_o_* = 0.240, *H_e_* = 0.264), also displaying a positive inbreeding coefficient and a minor heterozygote deficit (*F_IS_* = 0.076). The separate offspring population (N = 100) showed *I* = 0.391 and *H_e_* = 0.257 but a lower *H_o_* = 0.198, resulting in a higher inbreeding coefficient (*F_IS_* = 0.198), which indicates a more pronounced deficiency of heterozygotes.

The analysis of molecular variance (AMOVA) based on the 175 adult individuals revealed that 87% of the total genetic variation resided within subpopulations, whereas 13% was attributed to differences among subpopulations (Table 2). Genetic differentiation between the two adult subpopulations was significant (*Φ_PT_* = 0.131, *p* = 0.001).

### 2.3. Fine-Scale Spatial Genetic Structure

**Figure 2:**
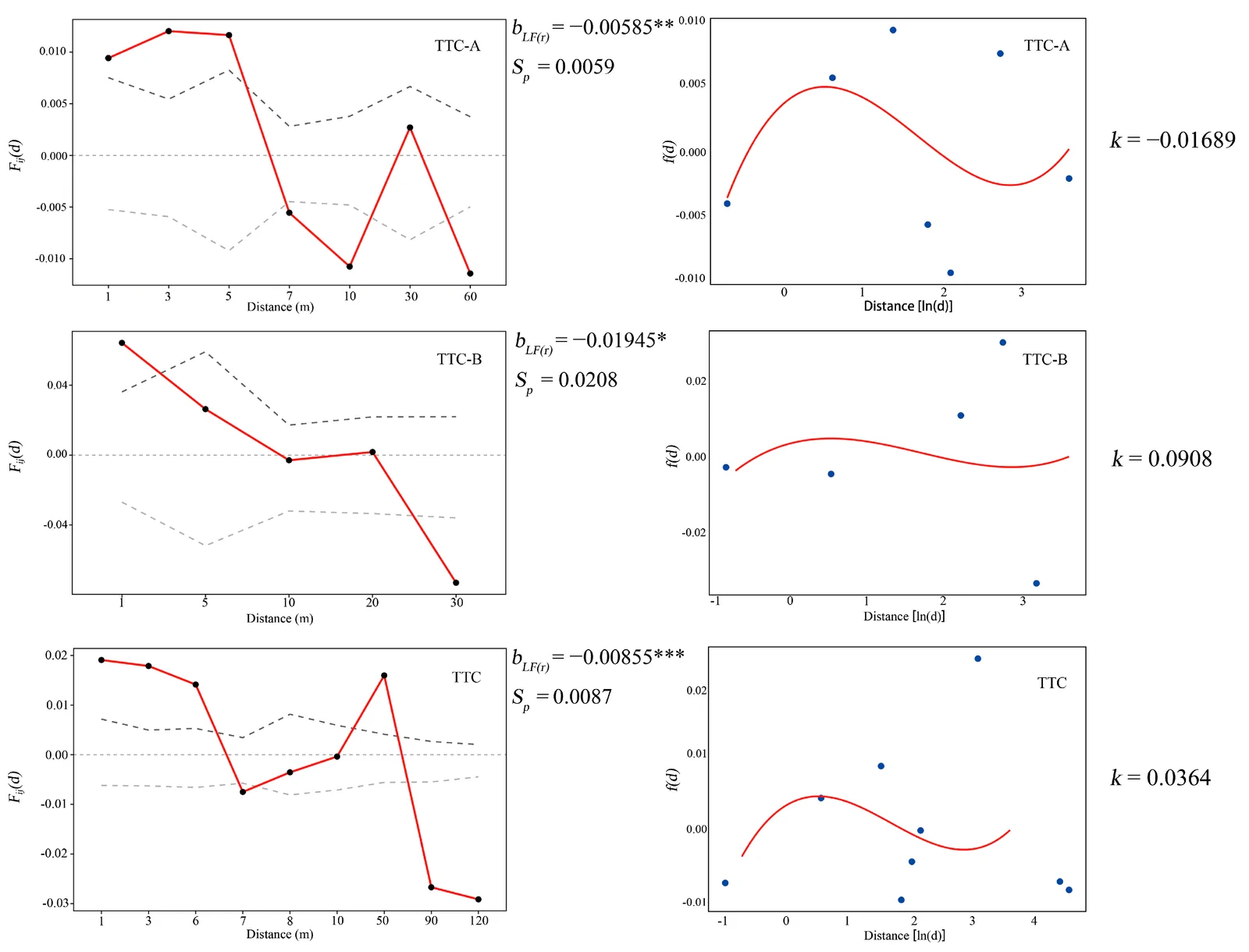
The left side shows the fine-scale spatial genetic structure of *R. gigantea*. The red line represents the average kinship coefficient F_ij_(d) between individuals within each distance class; grey dashed lines indicate the upper and lower bounds of the 95% confidence interval; bLF(r) represents the regression slope of the kinship coefficient F_ij_(d) against the natural logarithm of distance ln(d). Significance levels: * *p* < 0.05, ** *p* < 0.01, *** *p* < 0.001. The right side shows the three-variable linear regression analysis graph.

As shown in Figure 2, *R. gigantea* exhibited FSGS both across the entire area (TTC) and within subpopulations TTC-A and TTC-B. In TTC-A, significant FSGS was detected within approximately 5 m (*S_p_* = 0.0059), where individuals displayed relatively high genetic similarity; beyond this distance, the distribution became random, and at distances of around 30 m, a tendency toward uniform distribution was observed. In TTC-B, strong FSGS occurred within about 1 m (*S_p_* = 0.0208), followed by a trend toward uniform distribution as geographic distance increased. Across the entire area (TTC), significant FSGS was observed within 6 m (*S_p_* = 0.0087), with an additional signal of FSGS detected at d = 50 m. The ternary linear regression analysis of *f(d)* against log-transformed distance *ln(d)* further revealed curvature (*k*) values from the second derivative of the fitted curves: 0.0364 (TTC), −0.01689 (TTC-A), and 0.0908 (TTC-B). These results indicate that in the entire population (TTC, *k* > 0) and the TTC-B subpopulation (*k* > 0), seed dispersal was more restricted than pollen dispersal, whereas in the TTC-A subpopulation (*k* < 0), pollen dispersal was more limited than seed dispersal (Figure 2).

### 2.4. Mating System and Pollen Flow

The paternity analysis of *R. gigantea* using COLONY showed that the offspring of each capsule were assigned to a single full-sib family, indicating that seeds within a capsule were primarily sired by a single male parent (Table 3). All 10 seedlings within each individual capsule were assigned to a single full-sib family, indicating an absence of intra-capsule multiple paternity. However, when examining cross-capsule paternity variance on the same maternal individual, patterns diverged: both capsules from maternal plant TTC-7-6 (T7-6-1 and T7-6-2) shared the identical paternal source (selfed by TTC-7-6), demonstrating repeated single-donor pollination events across flowers. In contrast, the two distinct capsules sampled from maternal plant TTC-6-8 (T6-8-2 and T6-8-5) were sired by different fathers (TTC-6-11 and TTC-6-7, respectively), demonstrating cross-capsule paternity variance and independent outcrossing events on the same maternal host. Nine out of the ten analyzed capsules were assigned to candidate fathers with high confidence (assignment probability = 1.00). Following our strict confidence threshold (≥95%), the paternal origin of capsule T4-50-2 was classified as unassigned (candidate TTC-3-16 had a probability of only 0.41 and was rejected). Among the ten capsules analyzed, four were selfed (T4-85-1, T6-9-3, T7-6-1, and T7-6-2), whereas five were assigned as outcrossed. These results represent the mating outcomes of the successfully germinated capsules and should not be interpreted as the mating pattern of all 24 naturally pollinated capsules. Estimated pollen dispersal distances ranged from 0.30 m to 5.84 m, with a mean of 1.02 m, indicating predominantly short-distance pollen movement, although occasional longer-distance dispersal was observed.

## 3. Discussion

### 3.1. Genetic Diversity of Adult Plants and Progeny Populations

Understanding the divergence in genetic diversity and inbreeding coefficients between mature adults and early-stage progeny cohorts is critical for elucidating population regeneration dynamics and life-stage-specific selection pressures [5,32,33]. In this study, we observed a striking intergenerational genetic shift: whereas the contemporary F1 seedling cohort displayed a strong deficiency of heterozygotes (*F_IS_* = 0.198, *H_o_* = 0.198 < *H_e_* = 0.257) driven by high rates of geitonogamous self-pollination (44.4%), the mature adult population maintained near-equilibrium heterozygosity with a minimal inbreeding coefficient (*F_IS_* = 0.082, *H_o_* = 0.243 ≈ *H_e_* = 0.265) (Table 1).

This substantial reduction in inbreeding coefficients from the offspring cohort to the adult stage provides compelling, textbook evidence of intense post-zygotic selection and strong inbreeding depression operating across life-history transitions [5]. In *R. gigantea*, pollinator foraging behavior within large, multi-flowered inflorescences frequently mediates geitonogamy, generating heavily homozygous and inbred seed crops [31]. However, selfed progeny in orchids commonly harbor high genetic loads of deleterious recessive alleles, resulting in low embryo viability, protocorm abortion, reduced mycorrhizal colonization ability, and elevated seedling mortality [9,31]. Consequently, it is plausible that heavily homozygous, inbred individuals may experience selective disadvantages during early establishment stages, such that more outcrossed and heterozygous genotypes are more likely to successfully recruit into the reproductive adult cohort [12,34].

This life-stage selective filtering explains why long-lived adult epiphytes can maintain moderate genetic diversity and avoid severe inbreeding accumulation despite elevated contemporary selfing in fragmented forest remnants [4,20,33].

Importantly, because the F1 cohort in this study was reared via in vitro asymbiotic germination under sterile laboratory conditions, their observed genetic structure (*F_IS_* = 0.198) was completely independent of wild post-dispersal germination filters or mycorrhizal fungal constraints [35]. Instead, this offspring heterozygote deficiency was driven exclusively by pre-zygotic and contemporary pollination dynamics on maternal plants—specifically, high rates of insect-mediated geitonogamous self-pollination across large inflorescences. Consequently, the F1 cohort directly captures the immediate genetic output of local pollinator foraging behavior prior to the onset of natural post-dispersal environmental and fungal selection in the wild.

In this study, the two adult subpopulations of *R. gigantea* were separated by agricultural fields and exhibited significant genetic differentiation (*Φ_PT_* = 0.131, *p* = 0.001, Table 2). This result suggests that, although both seeds and pollen possess certain dispersal capabilities, agricultural land as a non-habitat landscape matrix restricts gene flow between subpopulations [36].

### 3.2. Detection of Fine-Scale Spatial Genetic Structure

FSGS reflects the relationship between genetic similarity among individuals and spatial distance, and serves as an important tool for understanding seed dispersal, pollen-mediated gene flow, and population regeneration dynamics [37]. In this study, significant FSGS was detected in *R. gigantea* at both the overall population level and within the two subpopulations. The estimated *S_p_* values fell within the range commonly reported for perennial woody plants and epiphytic species, indicating pronounced short-distance genetic clustering among individuals [22,23].

In contrast to the lab-reared F1 progeny, the FSGS observed among wild adult individuals is shaped by post-dispersal ecological recruitment bottlenecks under natural conditions. Although orchid seeds possess dust-like morphology facilitating potential long-distance wind dispersal [7], their natural germination in the canopy strictly requires compatible mycorrhizal fungi [9,10]. Because compatible fungal symbionts are often spatially aggregated around established adult host trees (functioning as ‘nurse sites’), successful wild recruitment is localized near maternal plants [8,24]. This fungal dependency, coupled with restricted seed movement within tree canopies, constrains effective seedling establishment and reinforces the strong adult spatial genetic clustering observed within 5–6 m across the study area.

At the overall population level (TTC), *R. gigantea* exhibited significant FSGS within a spatial scale of 6 m (*S_p_ =* 0.0087) (Figure 2). In addition, genetic clustering was detected at approximately 50 m, suggesting that genetic associations may persist at broader spatial scales due to ongoing gene flow or the spatial distribution pattern of host trees [21,22]. Notably, strong FSGS was observed within 1 m in the TTC-B subpopulation (*S_p_ =* 0.0208). Trivariate linear regression analyses further revealed differences in the relative constraints imposed by pollen and seed dispersal among populations. In the TTC-A subpopulation, pollen dispersal appeared to be more restricted than seed dispersal (*k* < 0), whereas seed dispersal was relatively more limited in the TTC-B subpopulation and the overall population (*k* > 0). These findings indicate that even populations within the same landscape may exhibit substantially different reproductive dispersal patterns and spatial genetic structures [38,39].

Such variation may result from subtle differences in habitat conditions between the two subpopulations. Within the study area, TTC-A is located along a roadside and close to human settlements, experiencing stronger anthropogenic disturbance and relatively sparse vegetation. In contrast, TTC-B is located farther from roads and settlements, with lower disturbance intensity, higher vegetation density, and greater canopy cover. These habitat differences may substantially influence gene flow patterns in orchids. In TTC-A, intensive human disturbance and sparse vegetation may disrupt pollinator activities, whereas the relatively open environment may facilitate seed dispersal by reducing physical barriers. Conversely, in TTC-B, higher vegetation density may constrain wind-mediated seed dispersal, while a relatively stable pollination environment may provide more favorable conditions for pollinator visitation and effective pollen transfer [40].

### 3.3. Paternity Analysis and Mating Systems

Selfing has been shown to significantly reduce seed developmental quality and germination potential in orchids [39,41,42]. Selfing increases offspring homozygosity, and when selfed or inbred offspring survive and accumulate near maternal plants or neighboring individuals, they can enhance genetic similarity among spatially adjacent individuals. Even when pollen dispersal occurs over moderate distances, the reduced viability of selfed seeds may prevent these offspring from successfully establishing stable populations at greater distances. Consequently, potential genetic inputs mediated by pollen flow may not be translated into long-term genetic mixing, thereby maintaining or even reinforcing fine-scale spatial genetic structure primarily driven by limited seed dispersal [12].

Seed viability and embryo development assessments in *R. gigantea* further support this interpretation, showing that outcrossed seeds possess significantly higher quality than selfed seeds, while selfed progeny exhibit pronounced developmental disadvantages during early life stages [31]. Evidence from both parentage analysis and seed viability assessments indicates that selfing not only compromises offspring survival and development but also shapes the spatial genetic composition of subsequent generations, thereby directly contributing to the formation of fine-scale spatial genetic structure in *R. gigantea*.

According to Chen (2025), *R. gigantea* exhibits a mixed mating system characterized by predominant outcrossing and partial self-compatibility [31]. In the present study, parentage analysis revealed an effective post-germination selfing rate of 44.4% (four out of nine resolved capsules) within the successfully germinated subset (Table 3). This indicates that geitonogamous self-pollination occurs frequently under natural conditions, aligning with previous observations of self-compatibility in *R. gigantea* [31] (Table 3). Furthermore, full-sib family assignment demonstrated that all offspring within each capsule belonged to a single full-sib family, confirming that each capsule was fertilized by a single pollen donor. This is consistent with the characteristic pollination biology of Orchidaceae, where pollen grains are aggregated into cohesive pollinia, typically resulting in single paternity for all ovules within a given flower, although the possibility of multiple pollinia transfers from different donors cannot be completely excluded across broader reproductive contexts. Notably, our results reveal clear patterns of cross-capsule paternity variance across different flowers on the same maternal plant, while maintaining strict single paternity within each individual capsule. Specifically, two distinct capsules from maternal plant TTC-7-6 (T7-6-1 and T7-6-2) shared an identical paternal origin, indicating repeated geitonogamous pollination by the same donor across separate flowers. Conversely, two capsules from maternal plant TTC-6-8 (T6-8-2 and T6-8-5) were sired by two distinct paternal donors (TTC-6-11 and TTC-6-7, respectively) (Table 3). This cross-capsule variation confirms that while each pollination event deposits a discrete pollinium, siring an entire fruit, distinct flowers on a single *R. gigantea* inflorescence are independently visited by different insect vectors carrying pollen from multiple donors across the canopy. These findings suggest that multiple flowers within the same maternal plant can be repeatedly pollinated by the same male donor, reflecting pollinator foraging behavior among different flowers within an individual plant. Although selfing may provide reproductive assurance under conditions of limited pollinator availability, the pronounced fine-scale spatial genetic structure observed in *R. gigantea* indicates that neighboring individuals are often genetically more similar. Elevated selfing rates may further increase mating among related individuals, resulting in greater local genetic homogenization and reinforcing genetic isolation. This self-reinforcing process may accelerate the erosion of local gene pools and reduce effective population size, ultimately threatening long-term population persistence.

The high effective selfing rate observed in *R. gigantea* is likely associated with its massive floral display and the distinct foraging behaviors of its insect visitors [31]. Field observations on Hainan Island indicated that large solitary carpenter bees (*Xylocopa* spp.) and Asian honeybees (*Apis cerana*) represent the primary floral visitors capable of removing and transferring pollinia [31]. We hypothesize that these two pollinator taxa may play divergent roles in shaping gene flow dynamics: on one hand, large-bodied *Xylocopa* bees possess strong flight capacities and could potentially facilitate outcrossing and pollen movement between host trees; on the other hand, *Apis cerana* workers frequently exhibit high floral constancy and tend to systematically probe multiple adjacent flowers within the same pendulous inflorescence before departing [31,41]. It is plausible that such prolonged residence time and intra-inflorescence foraging by honeybees could substantially elevate geitonogamous self-pollination. These non-mutually exclusive behavioral possibilities provide a tenable explanation for both the localized mean pollen dispersal distance (1.02 m) and the high proportion of selfed capsules detected in our parentage analysis, although direct tracking of individual pollinator flights will be needed to verify their relative contributions.

Experimental evidence supports this mechanism. In the deceptive orchid *A. morio*, nectar supplementation significantly increased the number of visited flowers, pollinator residence time, and the frequency of geitonogamous pollination events [41]. Moreover, studies on reward-based orchids, such as species of *Habenaria*, have shown that although nectar rewards enhance pollination efficiency, pollen dispersal distances remain relatively short, suggesting that reward systems primarily promote local genetic exchange [43]. In *R. gigantea*, the presence of large inflorescences may similarly increase pollinator visitation frequency while simultaneously increasing the probability of pollen transfer among flowers within the same inflorescence or between adjacent inflorescences, thereby elevating the likelihood of selfing.

A crucial caveat in our mating system estimation is the substantial germination bottleneck observed during F1 sampling. Out of 24 naturally pollinated capsules collected from 22 maternal plants, 14 failed to germinate entirely (a 58.3% germination failure rate), leaving only 10 viable capsules for genetic analysis. In self-compatible orchids, early-acting lethal inbreeding depression and high genetic loads frequently trigger complete embryo abortion and seed developmental arrest in selfed fruits [31,34,42]. The observed 44.4% selfing rate (or 40.0% of total germinated capsules) therefore represents an effective post-germination selfing rate that is inherently biased toward viable, outcrossed genotypes. The true rate of self-pollination at initial ovule fertilization under natural canopy conditions was almost certainly higher before early-stage lethal filtering occurred.

## 4. Materials and Methods

### 4.1. Study Species

During the summer of 2024, we conducted a comprehensive survey of all *R. gigantea* individuals in the study area, mapped their spatial distribution, and collected biological samples. The entire area was repeatedly surveyed to ensure comprehensive sampling coverage. Fresh leaf tissues were collected from 175 sexually mature adult individuals, classified based on size and immediately preserved in silica gel as parental materials for subsequent analyses.

**Figure 3:**
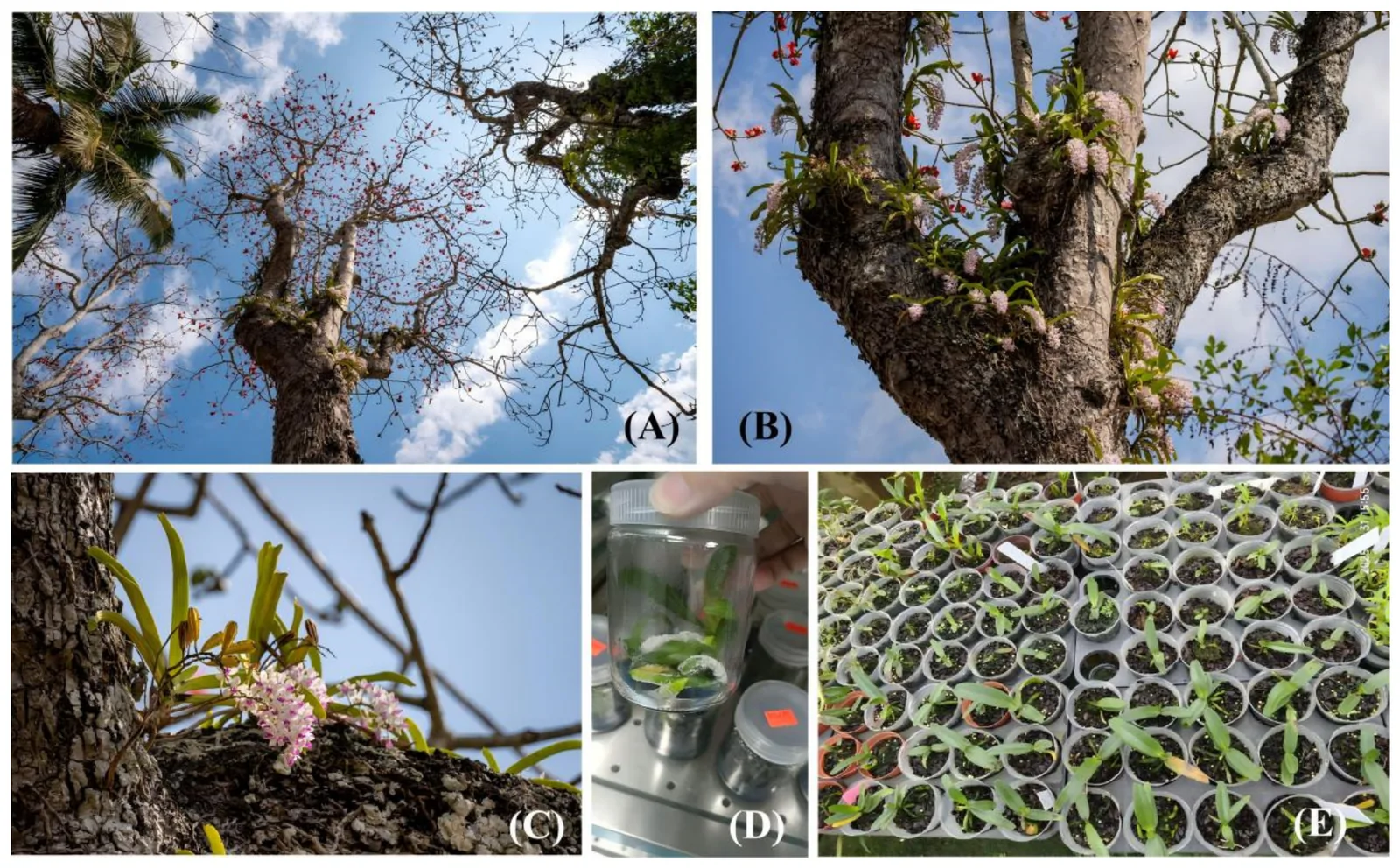
Ecological habitat, host trees, and plant materials of *R. gigantea.* (**A**) The host tree *Bombax ceiba* of *R. gigantea*; (**B**) *R. gigantea* individuals growing epiphytically on *B. ceiba*; (**C**) fruiting individuals of *R. gigantea*; (**D**) F1 seedlings of *R. gigantea* grown in Petri dishes; (**E**) F1 offspring of *R. gigantea*.

In December of the same year, 24 naturally pollinated capsules were collected from reproductive adult individuals, representing 22 different maternal plants. Following asymbiotic seed germination, only 10 capsules successfully germinated. These 10 successfully germinated capsules originated from seven maternal plants, with three maternal plants contributing two capsules each and four maternal plants contributing one capsule each. Ten seedlings were randomly selected from each successfully germinated capsule, resulting in a total of 100 F1 offspring individuals used for subsequent analyses (Figure 3). No protocorm splitting, clonal propagation, or vegetative multiplication was performed before DNA sampling.

The parental individuals of *R. gigantea* used in this study were collected from wild natural populations in Jiangbian Township, Dongfang City, Hainan Province, China (108°56.671432′ E, 18°54.061295′ N). The formal botanical identification of the specimens was confirmed by the research team at Hainan University. No commercial or externally sourced plant materials were used in this study.

### 4.2. Spatial Coordinate Measurement of Plants

To accurately characterize the three-dimensional spatial positions of *R. gigantea* individuals, we conducted systematic spatial measurements of all host trees and orchid individuals within the study plot [22]. All host trees with a DBH ≥ 1 cm were mapped to characterize the spatial distribution of woody vegetation and establish the spatial reference framework for positioning *R. gigantea* individuals. This threshold was used for spatial vegetation mapping and does not imply that *R. gigantea* commonly occurred on trees of this minimum diameter.

The spatial coordinates of host trees were measured using a total station theodolite. The base of each tree trunk was used as the reference point, and the electronic distance measurement (EDM) and angular measurement functions of the total station were used to obtain the x, y, and z coordinates of each host tree. Multiple base points were established throughout the study plot to ensure measurement accuracy and spatial coverage. The total station automatically calculated the relative spatial coordinates of each measurement point based on triangulation principles and three-dimensional spatial surveying algorithms.

Because *R. gigantea* individuals are epiphytic and attached to tree trunks or branches, their spatial coordinates were determined using a plumb-line vertical projection method. Specifically, the ground projection point of each epiphytic individual was established using a vertical plumb line, and its horizontal coordinates (x, y) were precisely surveyed using a total station. The vertical attachment height (z) above the ground was measured concurrently and combined with the ground coordinates to construct a complete three-dimensional coordinate dataset (x, y, z) for all sampled individuals. Pairwise three-dimensional Euclidean distances were then calculated from these coordinates for FSGS analyses. This approach explicitly accounts for both horizontal spatial separation and vertical stratification across host trees in shaping spatial genetic patterns.

### 4.3. DNA Extraction, SNP Calling, and Genotyping

Total genomic DNA was extracted from all samples using a modified CTAB method [21]. DNA integrity was assessed via 1.2% agarose gel electrophoresis, and concentration was quantified using a Qubit 3.0 fluorometer. Severely degraded samples were quantified based on the brightness of the main DNA band. Qualified samples were normalized to a final volume of 10 μL at 200 ng total DNA per sample. A double-digest RADseq (ddRAD) protocol was applied. Each DNA sample was digested with *EcoR I* and *Msp I* (NEB), followed by incubation at 37 °C for 8 h and 65 °C for 20 min, then held at 12 °C. Digestion efficiency was verified via agarose gel electrophoresis. Barcoded *EcoR I* adapters and common *Msp I* adapters were ligated using T4 DNA ligase (NEB) at 16 °C for 8 h. After ligation, all samples were pooled in equimolar volumes and subjected to size selection (400–600 bp) using agarose gel extraction (Omega Gel Extraction Kit, Omega Bio-tek, Norcross, GA, USA). The resulting library was PCR-amplified and sequenced on the BGI T7 platform (Guangzhou Jierui Biotechnology Co., Ltd., Guangzhou, China) using PE150.

To ensure the quality of downstream analyses, raw sequencing data were quality-filtered using the *process_radtags* module in Stacks v2.68 [44]. Based on the sequencing depth statistics of each sample after demultiplexing, the R1 reads of individual samples were processed using the *ustacks* module for clustering and deduplication to form loci. Key parameters were set as follows: M = 5 (the maximum number of mismatches allowed between alleles in a heterozygous individual) and m = 2 (the minimum depth of coverage required to create a stack). All individual loci were then used to construct a catalog file using the *cstacks* module, with the parameter n = 5. The *sstacks* module was subsequently used to match SNPs, alleles, and tag information from each sample against the catalog file, generating the corresponding matches file. Next, the *tsv2bam* and *gstacks* modules were used for format conversion and preparation prior to SNP calling. The *populations* module was then run to call shared SNP loci across populations. The parameters are set as follows: SNPs are filtered to retain those present in at least 80% of the population, with a minimum allele frequency of ≥ 0.05 and a minimum sequencing depth of ≥ 10× for individual genotyping; the first SNP at each locus is selected for subsequent analysis.

### 4.4. Genetic Diversity and Genetic Differentiation

To assess the genetic diversity of *R. gigantea* within the region, SNP site information was first extracted from the VCF file using Plink v2.0 and then analyzed using GenAlEx v6.5 [45,46]. The statistical analyses of genetic diversity included indicators such as observed heterozygosity (*H_o_*), expected heterozygosity (*He*), the inbreeding coefficient (*F_IS_*) and Shannon’s information index (*I*). In addition, an AMOVA was performed on the two subpopulations (TTC-A and TTC-B) in the region to investigate the degree of genetic differentiation and the sources of genetic variation.

### 4.5. Spatial Genetic Structure

To investigate whether FSGS occurs within the *R. gigantea* population, we employed the spatial autocorrelation method implemented in SPAGeDI v1.4 [47]. The kinship coefficient between individuals (*F*_ij_) was regressed against the natural logarithm of their spatial distance ln(*r*_ij_), yielding the regression slope *b*_F_ [48]. In order to ensure a relatively uniform distance gradient and a balanced number of pairwise samples within each distance class, 9 distance classes were defined based on the spatial distribution range of *R. gigantea* in the study area with upper distance boundaries at 1, 3, 6, 7, 8, 10, 50, 90, and 120 m (intervals: 0–1, 1–3, 3–6, 6–7, 7–8, 8–10, 10–50, 50–90, and 90–120 m). In addition, the TTC-A subpopulation was divided into 6 distance classes with upper boundaries at 1, 3, 5, 7, 10, and 30 m (or specified up to 60 m; intervals: 0–1, 1–3, 3–5, 5–7, 7–10, 10–30, and 30–60 m for 7 classes), and the TTC-B subpopulation was divided into 4 distance classes with upper boundaries at 1, 5, 10, and 20 m (intervals: 0–1, 1–5, 5–10, and 10–30 m, with 30 m as the maximum distance cutoff). The kinship coefficient *F*_ij_ and its 95% confidence interval for each distance class were obtained through 9999 permutation simulations. If the mean *F*_ij_ lies above the upper limit of the 95% confidence interval, it indicates that genetically similar individuals are spatially clustered, suggesting the presence of a significant spatial genetic structure. If the mean *F*_ij_ falls within the 95% confidence interval, it suggests a random spatial distribution of individuals, indicating no spatial genetic structure. If the mean *F*_ij_ is below the lower limit of the 95% confidence interval, it indicates a uniform spatial distribution of individuals with more distant genetic relationships. The *S_p_* statistic reflects the intensity of fine-scale spatial genetic structure and is defined as *S_p_* = – *b*_LF(r)_ *F*_(1)_, where *b*_F_ is the regression slope, and *F*_(1)_ is the mean kinship coefficient within the first distance class.

To predict the relative contributions of pollen flow and seed flow to gene flow among different populations, a ternary polynomial regression analysis was applied. In this model, the residual values *f(d)* [*F_ij_(d) − F_ij_(d)_exp_*] were used as the dependent variable, while the logarithm of distance ln(*d*) served as the independent variable. The regression equation was defined as follows:

$$f(d)=a+b\ln(d)+c{\left[\ln(d)\right]}^{2}+d{\left[\ln(d)\right]}^{3}$$

where *F_ij_*(*d*)*_exp_* represents the theoretical dependent variable values obtained from the linear regression of *F_ij_*(*d*) against ln(*d*).

Taking the second derivative of the function *f*(*d*), we obtain the curvature of the equation as follows:

$$k=2c+6d\ln(d_{1})$$

where *d*_1_ presents the mean inter-individual distance within a given distance class. When *k* > 0, it indicates that seed dispersal is more restricted than pollen dispersal, whereas when *k* < 0, it suggests that pollen dispersal is more constrained, or seed dispersal is relatively unconstrained.

### 4.6. Paternity Analysis

We imported the selected SNPs into COLONY v2.0 to perform parentage and sibship reconstruction on the F1 generation population [49,50]. COLONY implements a full-likelihood approach to parentage analysis. In our configuration, offspring were assigned to known maternal plants, while all surveyed adult individuals within the population (including the maternal plants themselves) were included in the candidate father pool. To accommodate this structure in COLONY, the following run parameters were specified: a monoecious/hermaphroditic model (enabling maternal individuals to also serve as candidate pollen donors), allowing inbreeding/selfing (to detect geitonogamous self-pollination events), diploid species, and polygamous mating systems for both males and females (defined in COLONY as permitting multiple mating events per individual). The polygamous model was chosen because a maternal plant bears multiple flowers that can receive pollen from different donors. Furthermore, although orchids package pollen into discrete pollinia that typically result in single paternity per flower/capsule, the possibility of multiple pollinia deposition or pollen competition from different donors on the same stigma cannot be entirely ruled out; thus, allowing polygamy avoids imposing artificial mating constraints. Analyses were conducted using the full-likelihood method with medium run length, medium precision, and without updating allele frequencies. We accepted the assignment for both paternity and maternity if results met one of the following criteria: (1) all three runs were assigned to the same parent with a probability of 95% or more; (2) assigned to the same parent three times and two of the runs assigned above 95%; (3) two runs assigned the same parent, both above 95%, but another run failed to assign any candidate parent. Assignments were regarded as failed when the most likely male parents were in conflict among results of three replicate runs.

## Figures and Tables

**Table 1:** Genetic diversity of two adult populations and F1 populations of *R. gigantea.*

Adults	N	*I*	*H_o_*	*H_e_*	*F_IS_*	Offspring	N	*I*	*H_o_*	*H_e_*	*F_IS_*
TTC-A	153	0.410	0.245	0.265	0.088	TTC-A	40	0.377	0.217	0.247	0.107
TTC-B	22	0.404	0.240	0.264	0.076	TTC-B	60	0.404	0.179	0.266	0.288
Total	175	0.407	0.243	0.265	0.082		100	0.391	0.198	0.257	0.198

Note: (N) sample size; (*H_o_*) observed heterozygosity; (*H_e_*) expected heterozygosity; (*F_IS_*) inbreeding coefficient.

**Table 2:** AMOVA for the two sub-populations of *R. gigantea*.

Source	df	*SS*	Est. Var. (%)	Percentage (%)	*Φ* *_PT_*	*p*-Value
Among Pops	1	9859.574	218.576	13%	0.131	0.001
Within Pops	173	251,071.809	1451.282	87%		
Total	174	260,931.383	1669.858	1		

Note: df, degrees of freedom; *SS*, sum of squares; Est. Var., estimated variance component; Percentage (%), percentage of total genetic variation. *Φ_PT_* is an analogue of Wright’s *F_ST_* calculated via AMOVA in GenAlEx, representing the proportion of genetic variance among subpopulations relative to the total variance. Statistical significance was tested using 1000 permutations (999 random permutations plus the original dataset, *p* = 0.001).

**Table 3:** Paternity analysis of 100 F1 seedlings from seven maternal plants with known maternal genotypes at the 95% confidence level.

Maternal Plants	Capsules	No. of F1	Fullsib Family (Prob.)	Paternal Plants (Prob.)	No. of F1 Seedlings Assigned	Pollen Dispersal Distance (m)
TTC-3-33	T3-33-1	10	1 (0.99)	TTC-3-31 (1.00)	10	0.80
TTC-4-50	T4-50-2	10	2 (1.00)	Unassigned (<0.95)	0	/
TTC-4-50	T4-50-6	10	3 (1.00)	TTC-4-40 (1.00)	10	0.30
TTC-4-84	T4-85-1	10	4 (1.00)	TTC-4-84 (1.00)	10	0.00
TTC-6-3	T6-3-1	10	5 (0.99)	TTC-6-10 (1.00)	10	5.84
TTC-6-8	T6-8-2	10	6 (0.99)	TTC-6-11 (1.00)	10	0.54
TTC-6-8	T6-8-5	10	8 (0.99)	TTC-6-7 (1.00)	10	1.70
TTC-6-9	T6-9-3	10	9 (1.00)	TTC-6-9 (1.00)	10	0.00
TTC-7-6	T7-6-1	10	11 (1.00)	TTC-7-6 (1.00)	10	0.00
TTC-7-6	T7-6-2	10	11 (1.00)	TTC-7-6 (1.00)	10	0.00
Means						1.02

## Data Availability

The datasets generated and analyzed during the current study are available from the corresponding author upon reasonable request. The raw high-throughput sequencing data generated in this study have been deposited in the NCBI Sequence Read Archive (SRA) database under the BioProject accession number PRJNA1521077 (Submission ID: SUB16447622).
